# Outcomes of post-exposure vaccination by modified vaccinia Ankara to prevent mpox (formerly monkeypox): a retrospective observational study in Lyon, France, June to August 2022

**DOI:** 10.2807/1560-7917.ES.2022.27.50.2200882

**Published:** 2022-12-15

**Authors:** Yanis Merad, Alexandre Gaymard, Laurent Cotte, Thomas Perpoint, Dulce Alfaiate, Matthieu Godinot, Agathe Becker, Olivier Cannesson, Anne-Sophie Batalla, Fatima Oria-Yassir, Sophie Landré, Florence Morfin, Maude Bouscambert, Florent Valour, Florence Ader, Anne Conrad

**Affiliations:** 1 Département des Maladies infectieuses et tropicales, Hôpital de la Croix-Rousse, Hôpital Edouard Herriot, Hospices Civils de Lyon, Lyon, France; 2 Laboratoire de Virologie, Institut des Agents Infectieux, Hôpital de la Croix-Rousse, Hospices Civils de Lyon, Lyon, France; 3 Centre International de Recherche en Infectiologie (CIRI), Inserm U1111, Université Claude Bernard Lyon 1, CNRS, UMR5308, Ecole Normale Supérieure de Lyon, Univ Lyon, Lyon, France; 4 Centre Gratuit D’Information, de Dépistage et de Diagnostic (CeGIDD), Hôpital de la Croix-Rousse, Hospices Civils de Lyon, Lyon, France; 5 Centre Gratuit D’Information, de Dépistage et de Diagnostic (CeGIDD), Hôpital Edouard Herriot, Hospices Civils de Lyon, Lyon, France

**Keywords:** Modified Vaccinia Virus Ankara, Monkeypox, Mpox, Post-exposure, Vaccination

## Abstract

Modified vaccinia virus Ankara vaccine (MVA-BN; Bavarian Nordic) is recommended to contacts of mpox cases up to 14 days post-exposure but the effectiveness of this strategy is unknown. Among 108 adults (≥ 18 years old) who received one dose of MVA-BN after exposure to mpox, 11 (10%) cases of breakthrough mpox were observed. Sexual exposure was associated with the risk of breakthrough mpox (p = 0.0179). Samples taken from vaccinated breakthrough mpox cases had similar rates of infectious virus isolation than unvaccinated mpox cases.

Since April 2022, a worldwide mpox (formerly monkeypox) outbreak is ongoing and predominantly affecting America and Europe, two continents historically non-endemic for the disease [1]. Outbreak cases are mostly occurring among men who have sex with men (MSM), with a high proportion presenting anogenital lesions, suggesting transmission during sexual activities [2]. To control the epidemic, health authorities of many countries have recommended vaccination with modified vaccinia virus Ankara vaccine (MVA-BN; Bavarian-Nordic), a third-generation vaccine indicated for immunisation against both smallpox and mpox. Vaccination is considered either as a primary (pre-exposure) preventive means for people at high-risk of exposure or as a post-exposure preventive (PEPV) measure for contacts of individuals with mpox [3-6]. However, evidence for the effectiveness of these two prophylactic strategies is scarce. 

The present analysis focusses on outcomes of at-risk contacts of mpox cases vaccinated with a single MVA-BN dose given post-exposure. The study provides data on breakthrough mpox and investigates factors limiting the PEPV strategy.

## MVA-BN for post-exposure prophylaxis

In France, since 20 May 2022 (week 20), PEPV with MVA-BN has been recommended to ‘at-risk’ contacts of mpox cases, i.e. individuals having experienced direct contact (through injured skin or body fluids), indirect contact (through textiles or surfaces) or prolonged (≥ 3 hours at < 2 m) droplet exposure to a mpox case [5,7]. The recommended vaccine schedule comprises two doses separated from each other by ≥ 28 days, with the first dose given as early as possible and within 14 days after exposure to mpox, and the second one as soon as possible, depending on availability, after day 28 post first dose. At-risk contacts are identified through contact-tracing measures or are people who self-declare as such, when presenting spontaneously for vaccination after having been alerted by a mpox case (contact-warning).

**Figure 1:**
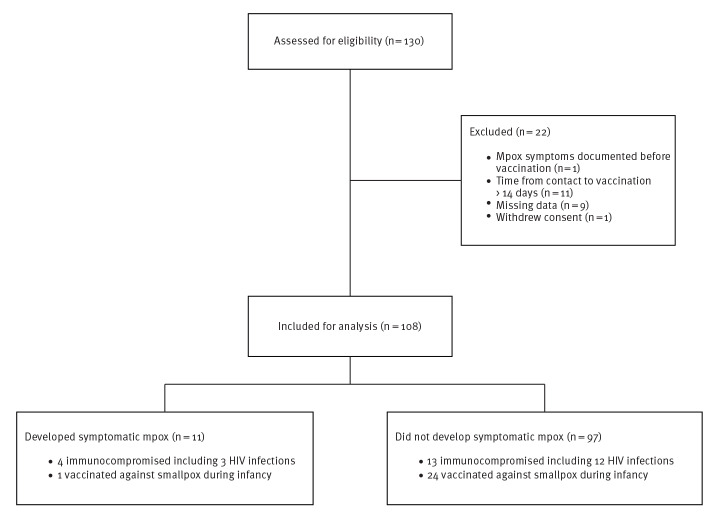
Flowchart of the study population selection, Lyon, France, June–August 2022 (n = 108)

Between 15 June and 12 August 2022, 130 adults (≥ 18-years old) consecutively received MVA-BN (IMVANEX) as part of PEPV at our hospital, the largest regional mpox testing and vaccination site in the Auvergne-Rhône-Alpes region. Of these, 108 individuals vaccinated with one dose of MVA-BN ≤ 14 days post-exposure (Figure 1) were included in our analysis. The study participants comprised 97 men (90%) and 11 women (10%) and had a median age of 35 years (interquartile range (IQR): 29–44). A total of 17 (16%) individuals were considered as immunocompromised, with 15 people living with HIV (including 1/15 with concomitant haematological malignancy), one solid organ transplant recipient and one patient receiving a TNFα inhibitor. Twenty-five (23%) individuals reported a history of smallpox vaccination during childhood.

Median time between exposure to the mpox case and reception of the first dose of MVA-BN was 9 days (IQR: 5–11), with 19 (18%) patients vaccinated within 4 days post-exposure. The main modes of exposure were sexual contact in 53 (49%) cases, cutaneous contact in 35 (32%) cases and indirect contact or respiratory droplets exposure in 20 (19%) cases.

## Breakthrough mpox

Breakthrough mpox was defined as symptomatic disease developing in a contact of a mpox case within 21 days after exposure despite PEPV. Occurrence of breakthrough mpox was considered as failure of the PEPV strategy. Breakthrough infections were identified during diagnostic consultations and through mandatory notifications of mpox to health authorities. Confirmation was obtained through detection of monkeypox virus (MPXV) DNA by real-time PCR on skin and/or mucosal swabs collected from lesions. As part of routine investigations, samples testing positive for MPXV by PCR were used to attempt viral isolation in cell culture, to assess virus infectivity. Details of laboratory methods are provided in the supplementary material. 

Eleven (10%) patients developed PCR-confirmed breakthrough mpox after vaccination with a median time between vaccination and symptom onset of 5 days (IQR: 1–6). Clinical course was mild and no patient required hospitalisation. Description of the population is provided in Table 1.

**Table 1:** Description of the study population and comparison between individuals developing or not mpox despite post-exposure preventive vaccination, according to univariate analysis, Lyon, France, June–August 2022 (n = 108)

Characteristics	Descriptive analysis^a^							Simple logistic regression analysis^b^	
	Total population (n = 108)		Developed breakthrough mpox (n = 11)		Did not develop breakthrough mpox (n = 97)		*p*-value	OR (95% CI)	p-value
	Number^c^	%^c^	Number^c^	%^c^	Number^c^	%^c^			
Demographic characteristics									
Male sex^d^	97	90	11	100	86	89	0.5990	NC	NC
Age in years^e^	Median (IQR)						0.2796	0.95 (0.88–1.01)	**0.1747**
	35 (29–44)		34 (28–36)		35 (29–45)				
Information related to immunosuppression or to prior smallpox immunisation^f^									
History of smallpox vaccination	25	23	1	NP	24	25	0.4512	0.30 (0.02–1.71)	0.2682
Immunosuppression	17	16	4^g^	NP	13^h^	13	**0.0696**	3.69 (0.87–14.12)	**0.0598**
HIV infection	15	14	3	NP	12	12	**0.1792**	2.66 (0.53–10.72)	**0.1891**
Type of exposure^i^									
Sexual	53	49	10	NP	43	44	**0.0037**	12.56 (2.27–234.90)	**0.0179**
Cutaneous	35	32	1	NP	34	35	**0.0994**	0.19 (0.01–1.03)	**0.1152**
Other close contact	20	19	0	NP	20	21	0.2113	NC	NC
Number of days from exposure to vaccination^j^	Median (IQR)						**0.1276**	0.86 (0.71–1.04)	**0.1200**
	9 (5.0–11.0)		7 (3.0–10.0)		9 (5.5–11.0)				

CI: confidence interval; NP: not presented due to the small sample size; NC: not calculable; OR: odds ratio.

^a^ Groups were compared using Fisher’s exact test or Mann–Whitney test, as appropriate.

^b^ OR (and their 95% CI) were estimated separately using simple logistic regression analysis.

^c^ Unless specified otherwise in certain cells of the table.

^d^ Information on sex was collected as a binary variable. ORs for male participants were not calculable.

^e ^Age was handled as a continuous variable and OR represents the change in odds by each one-year increase.

^f ^References for the ORs in this section were the absence of the characteristic in question (i.e. no smallpox immunisation, no immunosuppression, and no HIV).

^g^ Including three people living with HIV and one person receiving a TNFα inhibitor.

^h^ Including 12 people living with HIV (1/12 with concomitant haematological malignancy) and one solid organ transplant recipient.

^I ^The reference for the OR of each type of exposure in this section included all the other types of exposures combined.

^j ^Time from exposure to vaccination was handled as a continuous variable and OR represents the change in odds by each one-day increase.

p values < 0.2 are in bold type.

Swabs from clinical rash samples exhibited a median quantification cycle (Cq) value of 29.2 (range: 20.4–36). In 10 of 11 individuals with breakthrough mpox, virus infectivity was suggested by a positive viral culture, which could turn positive as early as during the first monitoring interval (i.e. 48–72 hours post inoculation). Overall, the median time for culture to become positive was 96 hours (range: < 72–168). Similar results were observed in non-vaccinated mpox patients (matched for age and HIV status, n = 11) followed at our centre, whose clinical samples had a median Cq value of 26.8 (range: 23–37.3); positive cell culture rate in this group was also 10/11 and median time to viral culture positivity was < 72 hours (range: < 72–240).

## Factors associated with failure of the PEPV strategy

**Figure 2:**
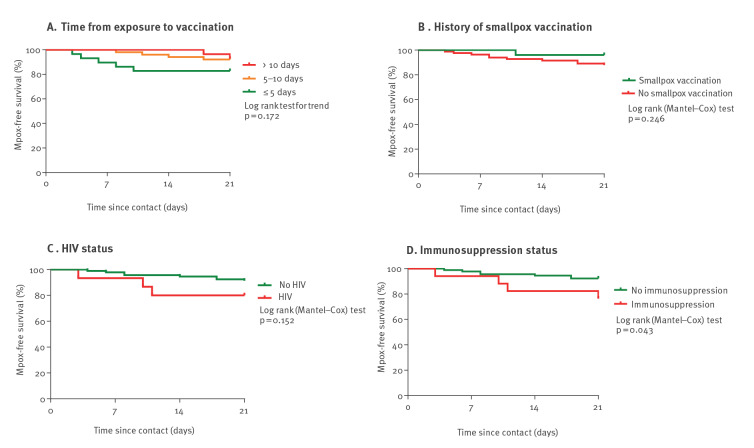
Kaplan–Meier plots of the probability of remaining mpox-free in the 21 days following exposure, according to (A) time from exposure to vaccination, (B) history of smallpox vaccination, (C) HIV and (D) immunosuppression status, Lyon, France, June–August 2022 (n = 108 contacts vaccinated post-exposure)

Potential risk factors of breakthrough mpox, i.e. failure of the PEPV strategy, were assessed using univariate analysis (Table 1). Sexual exposure (odds ratio (OR): 12.56; 95% confidence interval (CI): 2.27–234.90, p = 0.0179) was associated with breakthrough mpox. Immunosuppression (OR: 3.69; 95% CI: 0.87–14.12, p = 0.0598) tended to be associated with breakthrough mpox, however not for the subset of contacts with sexual exposure (OR: 2.20; 95% CI: 0.48–9.37, p = 0.2864) (Table 2). By plotting the probability of remaining mpox-free in the 21 days following exposure, on Kaplan–Meier curves (Figure 2A), it could be observed that patients developing breakthrough mpox tended to have been vaccinated earlier after exposure (within 5 days) than patients not developing breakthrough mpox (p = 0.172). This might suggest a higher awareness of the mpox risk and/or a more intensive exposure to mpox.

**Table 2:** Description of the subset of contacts with sexual exposure, and comparison between individuals developing or not mpox despite post-exposure preventive vaccination, according to univariate analysis, Lyon, France, June–August 2022 (n = 53)

Characteristics	Descriptive analysis^a^							Simple logistic regression analysis^b^	
	Sexual exposure (n = 53)		Developed breakthrough mpox (n = 10)		Did not develop breakthrough mpox (n = 43)		*p*-value	OR (95% CI)	*p*-value
	Number^c^	%^c^	Number^c^	%^c^	Number^c^	%^c^			
Demographic characteristics									
Male sex^d^	53	100	10	NP	43	100	> 0.9999	NC	NC
Age in years^e^	Median (IQR)						0.4209	0.95 (0.86–1.03)	0.2920
	34 (29–40.5)		32 (28–35.3)		34 (29–43)				
Information related to immunity or immunisation^f^									
History of smallpox vaccination	7	13	1	NP	6	14	> 0.9999	0.69 (0.03–4.76)	0.7407
Immunosuppression	14	26	4^g^	NP	10^h^	23	0.4258	2.20 (0.48–9.37)	0.2864
HIV infection	13	25	3	NP	10	23	0.6924	1.41 (0.27–6.21)	0.6563
Number of days from exposure to vaccination^i^	Median (IQR)						**0.0854**	0.83 (0.66–1.01)	**0.0811**
	9 (4.5–10.0)		6 (3.0–9.3)		9 (5.0–11.0)				

CI: confidence interval; NC: not calculable; NP: not presented due to the small sample size; OR: odds ratio.

^a^ Groups were compared using Fisher’s exact test or Mann–Whitney test, as appropriate.

^b^ OR (and their 95% CI) were estimated separately using simple logistic regression analysis.

^c^ Unless specified otherwise in certain cells of the table.

^d^ Information on sex was collected as a binary variable. ORs for male participants were not calculable.

^e ^Age was handled as a continuous variable and OR represents the change in odds by each one-year increase.

^f^ References for the ORs in this section were the absence of the characteristic in question (i.e. no smallpox immunisation, no immunosuppression, and no HIV).

^g^ Including three people living with HIV and one person receiving a TNFα inhibitor.

^h^ Including 10 people living with HIV (1/10 with concomitant haematological malignancy).

^I ^Time from exposure to vaccination was handled as a continuous variable and OR represents the change in odds by each one-day increase.

p values < 0.2 are in bold type.

All graphs represent Kaplan–Meier plots, which are a visual representation of the so-called ‘survival’ function. The y-axes represent the ‘survival’ probability (i.e. probability of remaining free of mpox) for contacts of mpox cases, and the x-axes represent the time elapsed since contact occurred. The curves are plotted for different groups of contacts (depending on when these groups were vaccinated against mpox, if they had prior received smallpox vaccination in their lives etc.). Groups are compared using the log-rank (Mantel–Cox) test.

## Discussion

In this investigation, PEPV by a single MVA-BN dose did not prevent symptomatic mpox in 10% of vaccinated contacts of mpox cases. Median time from vaccination to symptom onset was 5 days. Failure of the PEPV strategy was associated with sexual exposure.

Breakthrough mpox after vaccination has been observed before. For example, during the current outbreak, Hazra et al. reported 90 cases of mpox post-vaccination, of which 77% occurred 2 weeks after reception of a single dose of MVA-BN [8]. Their study nevertheless did not focus on PEPV. Our work, in contrast, provides information on the effectiveness of the PEPV strategy to prevent mpox. In another French cohort with PEPV, 4% of breakthrough mpox was noted among vaccinated contacts of cases [9]. Moreover, in the United Kingdom (UK), during an outbreak in 2018, one case of secondary mpox was detected in a healthcare worker despite PEPV with MVA-BN [10].

Prophylactic vaccination strategies to prevent mpox based on smallpox vaccines, take advantage of cross-protective immunity among orthopoxviruses. Epidemiological surveillance data from endemic areas in Africa in the past, has shown a 20-fold increase of mpox incidence following discontinuation of systematic smallpox vaccination, suggesting that smallpox vaccination could prevent mpox with an estimated vaccine effectiveness of 80–85% [11,12]. Similarly, during a mpox outbreak in 2003 in the United States, people who in their lives had been previously vaccinated against smallpox were less affected by mpox [13]. 

Prior to 2022, data on MVA-BN with regard to mpox prevention originated from animal challenge models, as well as animal and human immunogenicity studies [14-18]. The use of this vaccine in the current outbreak allows further insight on its capacity to protect people. In 2022, an analysis from the UK estimated that a single MVA-BN dose in high-risk MSM had an effectiveness of 78% against mpox [19]. In France, an important decrease of mpox incidence took place from week 27 2022, when pre-exposure vaccination (with at least one dose) of individuals at high-risk of exposure was implemented [6,20]. Up to 28 November 2022 a total of 138,383 doses of MVA-BN have been administered in the country [20].

While 10% of breakthrough mpox might seem a high proportion and was qualified in the current study as ‘failure’ of the PEPV strategy, this should be tempered for different reasons.

First, the objective of PEPV is not only to prevent symptomatic disease, but also to improve its course, protect against severe forms, and prevent further viral transmission by favouring viral clearance [14]. In the current outbreak, which has a low case fatality rate (49 deaths worldwide [1]), the potential of PEPV to alleviate clinical course is difficult to assess. On the other hand, we here provide virological data of breakthrough mpox. While in our study, Cq values and rates of virus isolation from clinical samples of non-vaccinated mpox patients were comparable to those of vaccinated individuals with breakthrough mpox, viral culture in the latter tended to turn positive later, suggesting slightly impaired infectivity; this preliminary observation, however, needs to be confirmed by future studies.

Second, as the full immunisation schedule comprises two doses (or even three doses in case of immunosuppression [6]) of MVA-BN, protection is expected to be incomplete after a single MVA-BN dose, especially in immunocompromised patients.

Finally, effectiveness of the PEPV strategy depends on the time elapsed between exposure and vaccine administration. While the optimal delay is ≤ 4 days, the median time between exposure and vaccine administration was 9 days in our cohort [3]. As incubation period of mpox has been estimated between 3 and 17 days, PEPV might intervene too late [3]. Interestingly, breakthrough mpox occurred more frequently in contacts vaccinated within 5 days post-exposure, even if this result did not reach statistical significance, likely due to a lack of power. While this may be counter-intuitive, it might be in fact a marker of high-risk exposure through sexual activities.

This study has some limitations: the retrospective design precluded collection of clinical details of breakthrough mpox. The proportion of breakthrough mpox, especially in sexually exposed contacts, might be underestimated because PEPV was largely offered to contact cases whatever the type of exposure, and because only symptomatic contacts were assessed, although data from the current outbreak suggest that 6.5% of asymptomatic MSM may have MPXV detected on anorectal swabs [21]. Finally, the analysis was conducted in a single centre and did not have a control group; moreover, the small sample size did not allow to draw any conclusions about women or transgender women and the number of cases was too low to perform accurate multivariate testing.

## Conclusion

While PEPV is at least partially effective to prevent mpox, association between sexual exposure and failure of the PEPV strategy strongly suggests that the PEPV strategy might be insufficient for patients at high-risk of exposure. Pre-exposure vaccination of this population could be more likely to break sexual transmission chains and contain the epidemic, as currently observed in Europe, where primary preventive vaccination strategies have been widely implemented.

## Supplementary Data

139000 Supplementary Material
