# Supplementary material for: Outcomes of post-exposure vaccination by modified vaccinia Ankara to prevent mpox (formerly monkeypox): a retrospective observational study in Lyon, France, June to August 2022
Source: Euro Surveill. 2022 Dec 15;27(50):2200882. doi: 10.2807/1560-7917.ES.2022.27.50.2200882 (PMC9808316; doi:10.2807/1560-7917.ES.2022.27.50.2200882)
Supplement: Supplementary Material [file 2200882_SupplementaryMaterial.pdf]

SUPPLEMENTARY DATA

This supplementary material is hosted by *Eurosurveillance* as supporting information alongside the article "Outcomes of post-exposure vaccination by modified vaccinia Ankara to prevent mpox (formerly monkeypox): a retrospective observational study in Lyon, France, June to August 2022", on behalf of the authors, who remain responsible for the accuracy and appropriateness of the content. The same standards for ethics, copyright, attributions and permissions as for the article apply. Supplements are not edited by *Eurosurveillance* and the journal is not responsible for the maintenance of any links or email addresses provided therein.

Virological methods

Every clinical sample was inoculated after one freeze thaw cycle on confluent Vero cells (ATCC©, CCL-81) seeded in 24 well plates with EMEM culture media, supplemented with 2% Penicillin Streptomycin + 1% L-Glutamine + 2% fetal bovine serum. The plates were incubated at 36°C under 5% CO_2 for 14 days. Cytotoxicity and virus growth were monitored by optical microscopy. To confirm the observation of cytopathic effects (CPE), the supernatant of each CPE positive well was confirmed by specific MPXV PCR. Time to culture positivity was determined when CPE were observed on at least 50% of the confluent cells.
All virus propagation experiments were performed in the BSL-3 laboratory in compliance with the Microorganism and Toxin (MOT) legislation at the *Institut des Agents Infectieux – Hospices Civils de Lyon*.

For diagnosis, we used a real-time PCR assay for the specific detection of monkeypox virus West African and Congo Basin strain [1]. This published PCR protocol was adapted for processing on the Hologic Panther Fusion (PF) system using its open access functionality. Briefly, 500µL of sample was transferred to an Aptima® multitest swab specimen collection tube (with a penetrable cap) containing 2.9mL Aptima® specimen transport medium (STM). Sample input for extraction was 360µL, elution volume was 50µL and template volume for each PCR reaction was 5µL. RNA/DNA extraction and amplification reagents (Open Access RNA/DNA enzyme cartridges, Extraction Reagent-S and Internal Control-S) were provided by Hologic (San Diego, CA, USA). A detailed description of the Primer/Probe Recon Solution and of the cycling conditions can be provided on request.

1. Li Y, Zhao H, Wilkins K, Hughes C, Damon IK. Real-time PCR assays for the specific detection of monkeypox virus West African and Congo Basin strain DNA. J Virol Methods. 2010 Oct;169(1):223–7.
